# Topoisomerase II inhibition involves characteristic chromosomal expression patterns

**DOI:** 10.1186/1471-2164-9-324

**Published:** 2008-07-08

**Authors:** Susanne Reymann, Jürgen Borlak

**Affiliations:** 1 Fraunhofer Institute of Toxicology and Experimental Medicine (Fh-ITEM), Center for Drug Research and Medical Biotechnology, Nikolai-Fuchs-Str. 1, 30625 Hannover, Germany; 2 Center of Pharmacology and Toxicology, Hannover Medical School, Carl-Neuberg-Str. 1, 30625 Hannover, Germany

## Abstract

**Background:**

The phenomenon of co-localization of transcriptionally upregulated genes showing similar expression levels is known across all eukaryotic genomes. We recently mapped the Aroclor 1254-regulated transcriptome back onto the genome and provided evidence for the statistically significant co-localization of regulated genes. They did, however, not always show similar expression levels, and many of the regulated genes were, in fact, repressed.

**Results:**

In this study, we were able to reproduce this observation with microarray data stemming from 1) human hepatocytes treated with the gyrase and potential topoisomerase II inhibitor trovafloxacin, 2) human hepatocytes treated with the topoisomerase II inhibitor doxorubicin and 3) mouse lymphoma cells treated with the topoisomerase II inhibitor etoposide. We found statistically significant co-localization of regulated gene pairs – induced and repressed – within the window size of 0–100 kbp. Notably, by using microarray data stemming from lung tissue of a mouse transgenic line overexpressing the transcription factor c-myc, which served as a negative control, we found regulated genes to be located with regard to each other nearly in the same way as genes distributed randomly all over the genome (0–100 kbp).

**Conclusion:**

We suggest topoisomerase II inhibition by Aroclor 1254, trovafloxacin, doxorubicin, and etoposide to be responsible for significant co-localization of regulated genes through the inability of the stabilized enzyme complexes to religate DNA. Within the permanently opened chromatin domains, neighbored genes might be allowed to be regulated. Overexpression of c-myc, however, does not inhibit topoisomerase II activity. Consequently, the enzyme is able to perform its normal function of transiently breaking and rejoining the DNA double strand. As a result, exclusively target genes are regulated.

## Background

Since the patterns of gene expression can be studied across the entire genome, it is known that binding of transcription factor complexes to regulatory sequences of genes is not the only mechanism controlling gene expression. It has often been shown that genomic location has an impact on gene expression [1]. Recently, it has been shown that identical reporter gene constructs integrated at 90 different chromosomal positions varied eightfold in their expression levels [2]. Furthermore, an intact gene can have a pathological phenotype at a novel genomic location [3]. Even before whole-genome sequences were available, some examples were known showing that genes are not randomly distributed along chromosomes. Co-localization and co-regulation have been shown for genes with related functions, as is the case for the rRNA, histone, Hox, and globin gene clusters. Later, Spellman and Rubin [4] described a transcriptional profiling study that revealed a surprising correlation between the organization of genes along Drosophila chromosomes and their expression levels. Specifically, co-localized genes, consisting of an average of 15 contiguous genes, show strikingly similar relative expression levels. These neighbored genes do not possess related functions. Moreover, the Human Transcriptome Map-integrated high-throughput expression data measured by SAGE (serial analysis of gene expression) revealed that the human genome consists of many domains/clusters of highly and weakly expressed genes [5]. Such domains/clusters have also been described in the mouse genome [6]. Highly and weakly expressed domains differs in gene density, GC content, and length of the introns, and this might be explained by a tendency for highly expressed housekeeping genes to cluster [7]. Thus, there is extensive evidence for the clustering of co-expressed genes across all major eukaryotic kingdoms. The physical range of these co-expressed gene clusters in mammals extends up to 1000 kbp.

We recently mapped the Aroclor 1254-regulated transcriptome back onto the genome in order to get insight into the large-scale regulation of transcription [8]. We found genes regulated by Aroclor 1254 – induced and repressed – to be located much closer to each other than genes distributed randomly all over the genome, and many regulated gene pairs were even found to be directly neighbored. This raised the possibility of the chromatin structure being involved in large-scale regulation of transcription. We, therefore, discussed a structural chromatin domain model in which distinct chromatin domains have been "opened" as a result of activation of a target gene. In these domains, the entire neighborhood has the potential of being expressed through the accessibility of the corresponding gene promoters for different transcription factors. This means that, besides being controlled individually, e.g. through AhR, genes may also be subject to regulation according to their location within the genome. Notably, increased chromatin accessibility is just as likely to facilitate the binding of repressors as activators, with the result that some genes would be upregulated and some downregulated. On the one hand, this is consistent with our Aroclor 1254 gene expression data, according to which both up- and downregulated genes were found in direct neighborhood. On the other hand though, this is not consistent with the neighborhood of co-regulation as described above.

Here, we mapped trovafloxacin- and doxorubicin-regulated transcriptomes of human hepatocytes and etoposide-regulated transcriptoms of mouse lymphoma cells back onto the genome and confirmed our previous results of chromosomal localization of the Aroclor 1254-regulated transcriptome. We found genes regulated by trovafloxacin, doxorubicin as well as by etoposide – induced and repressed – to be located much closer to each other than genes distributed randomly all over the genome (< 100 kbp). By mapping the transcriptome of lung tissue stemming from a mouse transgenic line overexpressing the oncogene c-myc back onto the genome, however, we found regulated genes to be located with regard to each other like genes distributed randomly all over the genome (< 100 kbp). Therefore, we suggest that topoisomerase II inhibition might be the reason for the chromosomal clustering of Aroclor 1254-, trovafloxacin-, doxorubicin-, and etoposide-regulated genes. This hypothesis explains the existence of up- and downregulated genes in the direct neighborhood and is consequently distinguishable from the observation of the neighborhood of co-regulation already described in the literature.

## Results

We intended to answer the question as to whether deregulated genes from other gene expression profiles than from Aroclor 1254-treated human hepatocytes [8] are also statistically significantly co-localized along the chromosomes. Therefore, we used 1) whole-genome microarray data of human hepatocytes treated with the gyrase and potential topoisomerase II inhibitor trovafloxacin, and 2) treated with the anthracycline antibiotic doxorubicin, 3) whole-genome microarray data of a mouse lymphoma cell line treated for with the topoisomerase II inhibitor etoposide which is used as a form of chemotherapy for different malignancies [9], and 4) whole-genome microarray data of lung tissue stemming from a mouse transgenic line overexpressing the oncogene c-myc. The complete Aroclor, c-myc, trovafloxacin and doxorubicin data have been deposited in NCBI's Gene Expression Omnibus (GEO) [10]. They are accessible through GEO Series accession numbers GSE5213, GSE10954 and the SuperSeries accession number GSE11942. The complete etoposide data are stored in EBI ArrayExpress [11] and are accessible through the accession number E-TOXM-5. The calculation of significant gene lists resulted in 927 deregulated genes for Aroclor 1254-treated human hepatocytes, 2241 deregulated genes for trovafloxacin-treated human hepatocytes, 784 deregulated genes for doxorubicin-treated human hepatocytes, 877 deregulated genes for etoposide-treated mouse lymphoma cells, and 1205 deregulated genes for lung tissue stemming from a mouse transgenic line overexpressing the oncogene c-myc. Lists of respective significantly deregulated genes possessing a RefSeq accession number were given in Additional File 1.

We calculated specifically the probability by which particular deregulated genes will occur within DNA windows of different sizes as compared to the probability of all known mapped genes (RefSeq transcripts) of the human genome (NCBI RefSeq 19,360; build 36.2) or of the mouse genome (NCBI RefSeq 13,517; build 36.1) to occur within the same DNA windows. RefSeq transcripts which possess the same gene symbol or the same start site of transcription and thus being alternatively spliced transcripts were included only once in the analysis, the other one was skipped.

The results of this analysis are depicted in Table 1. The numbers of observed pairs of deregulated genes in the different window sizes used and the mean of the numbers of pairs of genes stemming from the 100 random lists in the different window sizes used are listed in Table 1. This table gives also the standard deviations of the mean values of the random lists, as well as the significances (p-value), which express the possibility by which such a result would appear by chance. Significances were calculated by using the binominal distribution. Green labeling of values in Table 1 indicates overrepresentation of co-localized gene pairs within the window sizes 0–50, 50–100, or 100–200 kbp, whereas yellow labeling of values indicates underrepresentation of co-localized gene pairs within the window size 0–50 kbp. Most of the results were shown to be statistically highly significant. Thus, at least in the window sizes 0–50 and 50–100 kbp, genes which were regulated by Aroclor 1254 and doxorubicin and, moreover, in the window sizes 0–50, 50–100, and 100–200 kbp, genes which were regulated by trovafloxacin and etoposide were located much closer to each other than genes distributed randomly all over the genome. Different from this are the results for genes which were regulated by overexpression of c-myc. In the window size 0–50 kbp, genes are located much further from each other than genes distributed randomly all over the genome, whereas in the window size 50–100 kbp the distances of regulated genes are the same as of genes distributed randomly, and in the window size 100–200 kbp genes are also located much closer.

**Table 1:** Number of genes found in different window sizes.

	**Window sizes analyzed [kbp]**	**Expected number of genes (100 randomized gene lists)**	**Standard deviation**	**Observed number of regulated genes**	**Significance (p-value)**
Aroclor (599)	0–50	**15.07**	**3.23**	**34**	**0.00000857**
	50–100	**13.70**	**3.97**	**19**	**0.03657915**
	100–200	24.62	4.60	25	0.08103966
					
Trovafloxacin (1530)	0–50	**84.51**	**9.38**	**92**	**0.03045474**
	50–100	**77.16**	**7.71**	**110**	**0.00005667**
	100–200	**127.52**	**9.30**	**143**	**0.01301018**
					
Doxorubicin (580)	0–50	**13.33**	**3.61**	**21**	**0.01286883**
	50–100	**13.46**	**3.59**	**25**	**0.00140735**
	100–200	23.73	4.83	23	0.08357927
					
Etoposide (704)	0–50	**18.89**	**4.22**	**43**	**0.00024071**
	50–100	**18.50**	**4.44**	**46**	**0.00336279**
	100–200	**34.56**	**4.78**	**81**	**0.00065127**
					
c-myc (899)	0–50	*30.82*	*5.36*	*23*	*0.02745216*
	50–100	29.27	5.67	29	0.07500742
	100–200	**52.18**	**7.53**	**60**	**0.02919296**

Comparison of the gene lists resulting from treatment with different topoisomerase II inhibitors or from overexpression of c-myc with the mean of 100 randomized gene lists. For each window size, the expected number of genes is shown along with the observed number of genes. The expected numbers of genes result from the mean of 100 randomized gene lists comprising the same number of genes as the respective regulated gene list. Furthermore, the p-value is shown for obtaining such a result by chance using the binominal distribution. Whenever the difference between the expected number of genes and the observed number of regulated genes is significant, the corresponding values are marked bold (expected number < observed number) or italic (expected number > observed number).

**Figure 1:**
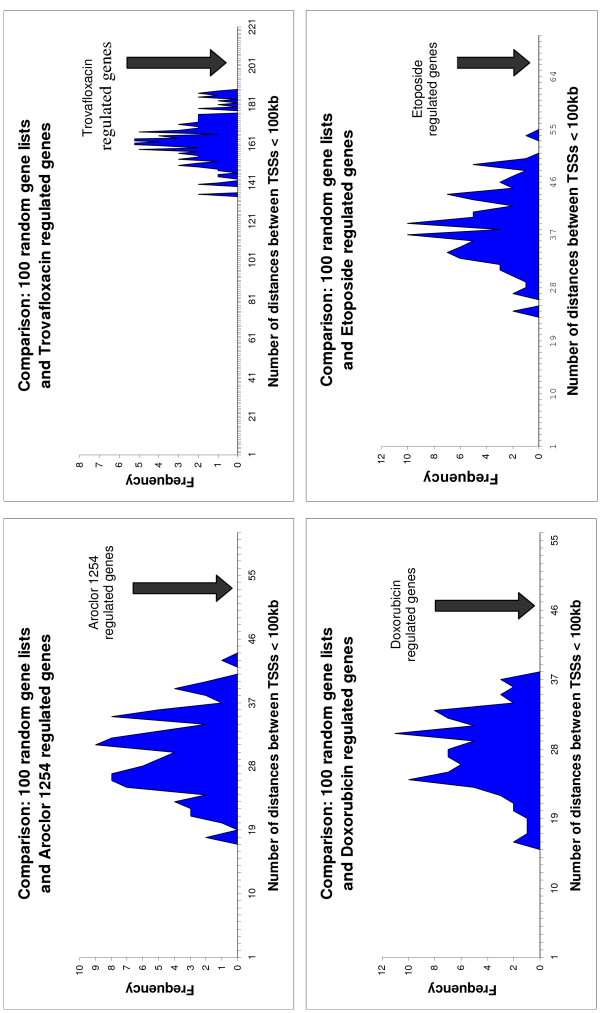
**Treatment with topoisomerase II inhibitors and potential topoisomerase II inhibitors: Frequency of the number of distances < 100 kbp between transcription start sites (TSSs)**. The number of distances < 100 kbp between TSSs was calculated for 100 randomized gene lists on the one hand and for the different significant gene lists stemming from human hepatocytes and mouse lymphoma cells treated with different topoisomerase II inhibitors and potential topoisomerase II inhibitors, respectively, on the other hand. The number of distances < 100 kbp between TSSs of the significantly regulated genes is marked by a black arrow in the corresponding histograms.

**Figure 2:**
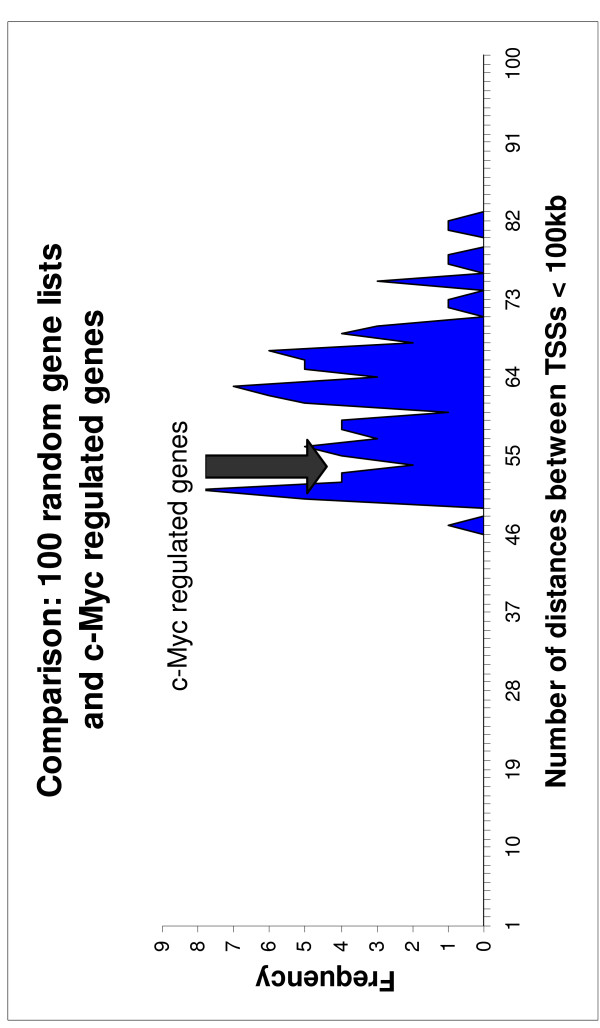
**Overexpression of c-myc: Frequency of the number of distances < 100 kbp between transcription start sites (TSSs)**. The number of distances < 100 kbp between TSSs was calculated for 100 randomized gene lists on the one hand and for the significant gene list stemming from lung tissue of a mouse transgenic line overexpressing the oncogene c-myc on the other hand. The number of distances < 100 kbp between TSSs of the significantly regulated genes is marked by a black arrow in the histograms.

The results are depicted graphically in Figures 1 and 2. The number of distances < 100 kbp between transcription start sites of two genes stemming from the 100 randomized gene lists were used to generate a histogram. In all five histograms, a Gaussian distribution with means of ~30 genes (Aroclor 1254), ~163 genes (trovafloxacin), ~27 genes (doxorubicin), ~39 genes (etoposide), and ~64 genes (c-myc) becomes apparent. In addition, the number of distances < 100 kbp between transcription start sites of two genes stemming from the respective significant gene list is marked in the histograms by black arrows. In the case of Aroclor 1254-, trovafloxacin-, etoposide-, and doxorubicin-regulated genes the strong significant deviation from the Gaussian distribution resulting from the 100 randomized gene lists is obvious (Figure 1). In the case of c-myc-regulated genes the number of distances < 100 kbp between TSSs is within the Gaussian distribution (Figure 2).

The regulated genes whose TSSs are located within 100 kbp and their corresponding expression values are marked yellow in Additional File 1. The file clearly shows that these gene pairs do not show similar expression levels. In many cases transcriptionally induced as well as transcriptionally repressed genes occur within 100 kbp.

## Discussion

Topoisomerases are ubiquitous enzymes that alter DNA topology by cleavage and religation of the DNA. Human topoisomerase II exists as two isoforms, known as topoisomerase IIα (174 kDa) and topoisomerase IIβ (182 kDa), which are essential for a number of nuclear processes, including chromosome condensation, chromatid separation, and relief of torsional stress during DNA replication and transcription [12]. Multiple enzymatic activities are required for the transcriptional initiation. The enzyme DNA topoisomerase II associates with gene promoter regions and generates breaks in double-stranded DNA (dsDNA), which are required for the activation of gene transcription by DNA-binding transcription factors [13]. These topoisomerases have been shown to be targeted by different topoisomerase poisons, as they stabilize the enzyme-mediated double-stranded breaks in the DNA [14].

In order to test for statistically significant chromosomal co-localization of regulated genes, we used different whole-genome microarray data. We used four different microarray data stemming from compound-treatment experiments: human hepatocytes treated with 1) Aroclor 1254, as published recently [8], with 2) trovafloxacin [15], with 3) doxorubicin, and with 4) etoposide [9]. These four compounds have a common characteristic: they may act as topoisomerase II poisons.

A variety of important anticancer drugs kill cells by increasing cellular levels of topoisomerase II-DNA cleavage complex [16]. The anthracycline anticancer drug doxorubicin [17] and potent anti-tumor drug etoposide [18] form a stable ternary complex with DNA and topoisomerase IIα, thereby inhibiting the normal function of the enzyme. The complexed enzyme is unable to religate DNA, so that complex formation increases DNA strand breaks. Most topoisomerase II poisons are believed to function at the active site of the enzyme via noncovalent interactions [16].

Recent studies, however, indicate that sulfhydryl-reactive quinones such as PCB quinones also have the potential to increase levels of topoisomerase II-mediated DNA cleavage [19-21]. It is believed that these compounds act by adducing amino acid residues that may lie outside the active site of the enzyme [19,20]. Aroclor 1254 is a well-known hepatotoxin, consisting of a complex mixture of polychlorinated biphenyls (PCBs) and thus may being a potent topoisomerase IIα poison as well [19].

Trovafloxacin is a broad spectrum antibiotic that inhibits the uncoiling of supercoiled DNA in various bacteria by blocking the activity of DNA gyrase and topoisomerase IV [15]. Specific members of this drug family display high activity against eukaryotic type II topoisomerase, as well as cultured mammalian cells and in vivo tumor models [22,23]. It has been discussed by Waring et al. [24], that trovafloxacin has a higher affinity for eukaryotic polymerase II system than the other quinolone agents tested. This effect coupled with other factors, such as an inflammatory response, might result in a hepatotoxic reaction seen with drug. Furthermore, Liguori et al. [25] performed microarray studies comparing etoposide with trovafloxacin and demonstrated similar gene expression changes occurring for topoisomerase II, RNA polymerase I and II, and other genes involved in transcription and RNA processing. Consequently, the authors suggested that trovafloxacin has a higher cross-reactivity with the eukaryotic polymerase II system than the other quinolone agents tested. Moreover, binding of quinolones to eukaryotic topoisomerases II has been suggested to contribute to cytotoxicity. Notably, trovafloxacin showed a cytotoxic effect in mammalian cells in vitro [26].

In eukaryotes, chromatin structure plays a major role in all aspects of DNA metabolism including transcription, replication, and repair. Changes in accessibility of DNA to nucleases in response to different stimuli reveal that its structure is dynamic. For instance, it is known that histone tail acetylation facilitates transcription, because acetylated histones restrain and stabilize negative supercoils less tightly than when unmodified [27]. Increased or decreased torsional strain may alter transcription and may antagonize or synergize with chromatin modification and remodeling [28].

Recently, Collins et al. [29] monitored changes in the basal transcriptional activity for many genes by using topoisomerase inhibitors. Examined genes displayed an individualized profile in response to inhibition of topoisomerases I and II. Expression changes elicited by camptothecin (topoisomerase I inhibitor) on the one hand and adriamycin/doxorubicin (topoisomerase II inhibitor) on the other hand were not equivalent. Camptothecin generally caused transcription complexes to stall in the midst of transcription units, while provoking little response at promoters. Adriamycin, in contrast, caused dramatic changes at or near promoters and prevented transcription. The authors have discussed different possibilities that might be involved in the downregulation of diverse genes by toposiomerase II inhibition, and assume that the distribution and transmission of torsional strain are disturbed. Furthermore, through the direct protein-protein interaction of topoisomerase II with transcription factors, the basal transcription apparatus, or chromatin-modifying or -remodeling machinery, the expression of specific genes might be altered. Furthermore, immobilized topoisomerase II-inhibitor-DNA complexes might form a blockade hindering the movement of transcription elongation complexes.

Moreover, another study [30] reported stimulation of IRF-7 gene expression by topoisomerase II inhibitors. The authors showed by chromatin immunoprecipitation assay that treatment with the topoisomerase II inhibitor etoposide induced association of aceteylated histone 3 with the promoter of IRF-7 gene, indicating that changes in the chromatin structure by the topoisomerase II inhibitor could be responsible for the induction. They further proposed that creation of DNA double strand breaks by topoisomerase II inhibitors results in relaxation of the chromatin structure at the IRF-7 promoter and exposure of the binding sites for the transcription factors already present in cells.

Only very few genes were regulated in common by Aroclor 1254, doxorubicin and etoposide. Between each two compounds, the numbers of genes regulated in common were 3.7% to 5.5% (see Additional File 1). Although these compounds may inhibit topoisomerase II they also excert a number of different reactions as well. Correspondingly, we would not expect to see more commonality in the regulated genes. In response to treatment with Aroclor, doxorubicin, or etoposide the enzyme DNA topoisomerase II associates with the corresponding target gene promoter regions and generates breaks in double-stranded DNA (dsDNA), to enable activation of gene transcription. The topoisomerases can be targeted by these agents and stabilize the enzyme-mediated double-stranded breaks in the DNA. The observed regulation of genes depends on regulatory sequences, targeted by transcription factors which can be activated directly or indirectly by Aroclor 1254, doxorubicin and etoposide therefore giving rise to individual transcript signatures. This agrees well with the fact that the different compounds show different side effects (e.g. doxorubicin shows primary cardiac side effects, including congestive heart failure, dilated cardiomyopathy, whereas Aroclor 1254 elicits skin effects such as chloracne and rashes, and liver damage).

Transcriptional consequences of topoisomerase II inhibition as transcriptional induction as well as transcriptional repression of different genes have been described in various studies. Chromatin domains, which are the units of transcriptional competence, are opened through DNA double strand breaks by topoisomerase II to reduce the torsional stress for subsequent transcription. Topoisomerase poisons stabilize the enzyme-mediated double-stranded breaks in the DNA [14]. These chromatin domains, however, remain opened, because the stabilized enzyme complex is unable to religate DNA. Through the direct protein-protein interaction of topoisomerase II with transcription factors, the basal transcription apparatus, or chromatin-modifying or -remodeling machinery, the expression of specific genes might be altered. Immobilized topoisomerase II inhibitor-DNA complexes might also form a blockade hindering the movement of transcription elongation complexes. Furthermore, other genes which are located also in the same opened chromatin domain now are accessible for transcription factors already present in cells. Strikingly, the size of chromatin domains, which are the units of transcriptional competences, varies from 5 kb to 200 kb [31]. This size corresponds to our results of statistically significant co-localization of gene pairs regulated by different topoisomerase II inhibitors within the window size of 0–100 kbp. This suggestion also corresponds to our findings of transcriptionally induced **and **repressed genes being located in direct neighborhood and does not contradict the phenomenon of co-localization of transcriptionally induced genes showing similar expression levels, which is known across all eukaryotic kingdoms.

Furthermore, we used microarray data stemming from lung tissue of a mouse transgenic line overexpressing the oncogene c-myc. C-myc is a multifunctional, nuclear phosphoprotein that plays a role in cell cycle progression, apoptosis, and cellular transformation. It functions as a transcription factor that regulates transcription of specific target genes. Here, topoisomerase II was not influenced and thus was able to perform its normal function of transiently breaking and rejoining the phosphodiester backbone of both strands of the double helix. Through transient opening of a chromatin domain, only a single target gene might be regulated. We thus found c-myc-regulated genes to be located with regard to each other nearly in the same way as genes distributed randomly all over the genome (< 100 kbp). This observation confirms our hypothesis of topoisomerase II inhibition involving statistically significant co-localization of regulated gene pairs – induced and repressed – within the window size of 0–100 kbp.

Taken together, the results presented in our study have revealed some interesting features of gene regulation in the presence of topoisomerase II inhibitors and potential topoisomerase II inhibitors. We suggest chromosomal co-localization of genes regulated in response to topoisomerase II poisons to be a direct consequence of topoisomerase II inhibition. Notably, genes which are located in the neighborhood of the target gene within a single chromatin domain are allowed to be regulated through the inability of the stabilized enzyme complexes to religate DNA. In contrast, in the case where topoisomerase II was not influenced (c-myc overexpression) the enzyme was able to perform its normal function of transiently breaking and rejoining the DNA double strand, so that only single target genes could be regulated.

## Methods

### Cell culture and tissue samples

Human hepatocytes were isolated from specimens obtained from patients undergoing hepatic resections as described by Borlak et al. [32]. They were cultured enclosed between two layers of collagen as described previously [32]. Prior to exposure, cells were allowed to recover from the isolation procedure for 4 to 5 days. Cells were harvested 72 hours after treatment with 100 μg/ml of trovafloxacin or 5 days after daily treatment with doxorubicin (0.1 μg/ml, 0.172 μM). Human hepatocytes were treated with Aroclor 1254 as described in Reymann et al. [8], and mouse lymphoma cells were treated with etoposide as described in Newton et al. [9]. C-myc-transgenic female mice displayed morphological alterations with varying degree of nuclear atypia, such as bronchiolo-adenomas and bronchiolo-adenocarcinomas. Different stages of malignant transformation of alveolar epithelium were observed. In the non-transgenic control animals no abnormalities in lung tissue were detected, with the exception of a single animal which showed a slight focal interstitial mononuclear cell infiltration.

### Gene expression studies

Transcriptome analyses of trovafloxacin- and doxorubicin-treated primary human hepatocytes and lung tissue of c-myc-transgenic female mice were done according to the manufacturer's recommendation (Affymetrix GeneChip^® ^Expression Analysis Technical Manual (Santa Clara, CA)), using the GeneChip^® ^Test Arrays, GeneChip^® ^Human Expression Array HG-U133 plus 2.0, and GeneChip^® ^Mouse Genome 430 2.0. Total RNA was isolated from the tissues using the RNeasy total RNA isolation kit (QIAGEN). A second cleanup of isolated RNA was done using the RNeasy Mini Kit (QIAGEN). RNA was checked for quantity, purity, and integrity of the 18S and 28S ribosomal bands by capillary electrophoresis using the NanoDrop ND-1000 and the Agilent 2100 Bioanalyzer. 1–15 μg of total RNA were used as starting material to prepare cDNA. Synthesis of double-stranded cDNA was done with the GeneChip^® ^one-cycle cDNA Kit (Affymetrix). The cleanup of double-stranded cDNA was done using the GeneChip^® ^Sample Cleanup module (Affymetrix). 12 μl of cDNA solution were used for *in vitro *transcription. The *in vitro *transcription was performed with the GeneChip^® ^IVT Labeling Kit (Affymetrix). The total amount of the reaction product was purified with the GeneChip^® ^Sample Cleanup module (Affymetrix). Purified cRNA was quantified and checked for quality using the NanoDrop ND-1000 and the Agilent 2100 Bioanalyzer. Purified cRNA was cleaved into fragments of 35–200 bases by metal-induced hydrolysis. The degree of fragmentation and the length distribution of the fragmented biotinylated cRNA were checked by capillary electrophoresis using the Agilent 2100 Bioanalyzer.

10 μg of biotinylated fragmented cRNA were hybridized onto the GeneChip^® ^array according to the manufacturer's recommendation. The hybridization was performed for 16 hours at 60 rpm and 45°C in the GeneChip^® ^Hybridization Oven 640 (Affymetrix). Washing and staining of the arrays was done on the GeneChip^® ^Fluidics Station 400 (Affymetrix) according to the manufacturer's recommendation. The antibody signal amplification, washing, and staining protocol (Affymetrix) was used to stain the arrays with streptavidin R-phycoerythrin (SAPE; Invitrogen, USA). To amplify staining, SAPE solution was added twice with a biotinylated anti-streptavidin antibody (Vector Laboratories, CA) staining step in between.

The arrays were scanned using the GeneChip^® ^Scanner 3000. Scanned image files were visually inspected for artifacts and then analyzed, each image being scaled to the same target value for comparison between chips. The GeneChip^® ^Operating Software (GCOS) was used to control the fluidics station and the scanner, to capture probe array data, and to analyze hybridization intensity data. Default parameters provided in the Affymetrix data analysis software package were applied for analysis. Transcriptome analyses of Aroclor 1254-treated primary human hepatocytes and etoposide-treated mouse lymphoma cells are described in Reymann et al. and Newton et al., respectively [8,9].

### Data analysis

Four of the datasets used have been deposited in NCBIs Gene Expression Omnibus (GEO) [10]. They are accessible through GEO Series accession numbers GSE5213 (Aroclor 1254), GSE10954 (c-myc) and the SuperSeries accession number GSE11942 (trovafloxacin and doxorubicin). The complete etoposide data are stored in EBI ArrayExpress [11] and are accessible through the accession number E-TOXM-5. Affymetrix .CEL files were uploaded into our database ArrayTrack [33], which is a toxicogenomics software for microarray data management and analysis. Significant gene lists were prepared in ArrayTrack by using the t-test, applying the following criteria: Aroclor 1254: as described in Reymann et al., 2006; trovafloxacin: p-values < 0.05, mean channel intensities > 100, bad flags <= 2, abs fold-change > 2.5; doxorubicin: p-values < 0.05, mean channel intensities > 50, bad flags <= 6, abs fold-change > 1.2; etoposide: p-values < 0.05, mean channel intensities > 80, bad flags <= 6, abs fold-change > 1.4; c-myc: p-values < 0.05; mean channel intensities > 80; bad flags <= 2; abs fold-change > 1.6.

### Analysis of chromosomal localization

From all lists of significantly regulated genes, lists of genes whose chromosomal positions are known and which possess a RefSeq accession number were created (Additional File 1). The start positions of transcription of all RefSeq transcripts of the human genome (= 19,360) and from the mouse genome (= 13,517) were downloaded from the NCBI genome browser MapViewer [34]. Out of this dataset, 100 random lists possessing the same number of RefSeqs as the corresponding significant gene lists were created. The numbers of pairs of genes for each of the lists that were located on the same chromosome within a distance of > 0–50, > 50–100, and > 100–200 (window sizes) were calculated. The numbers obtained from the lists of regulated genes were compared with the mean obtained from the 100 random lists. To determine if the observed (regulated) numbers of adjacent pairs were significantly different from those of expected adjacent pairs (100 random lists), the binominal distribution, given by the below formula was used.

$$B(k|p,n)=\left(\begin{array}{c} n\\ k \end{array}\right)p^{k}q^{n-k}$$

In this formula, *n *is the total number of RefSeq transcripts in the respective gene lists, *p *is the probability of a gene pair to be present in a single window size (expected number of gene pairs in a single window size/number of RefSeq transcripts in the respective gene lists), *k *is the observed number of gene pairs in a single window size, and *q *= 1-*p*.

## Authors' contributions

SR was responsible for the bioinformatical analysis of the study. JB initiated the study, and was responsible for the experimental part. Both authors drafted the manuscript.

## Supplementary Material

Additional File 1 **Lists of genes significantly deregulated after treatment with Aroclor1254, trovafloxacin, doxorubicin, etoposide and after overexpression of c-myc**. Exclusively genes possessing a RefSeq accession number were listed. The regulated genes whose TSSs are located within 100 kbp and their corresponding expression values are marked yellow. The file clearly shows that these gene pairs do not show similar expression levels. In many cases transcriptionally induced as well as transcriptionally repressed genes occur within 100 kbp. Click here for file
